# Supplementary figures and images for: Nutritional composition, fatty acids profile and immunoglobulin G concentrations of mare milk of the Chilean Corralero horse breed
Source: PLoS One. 2024 Sep 19;19(9):e0310693. doi: 10.1371/journal.pone.0310693 (PMC11412646; doi:10.1371/journal.pone.0310693)

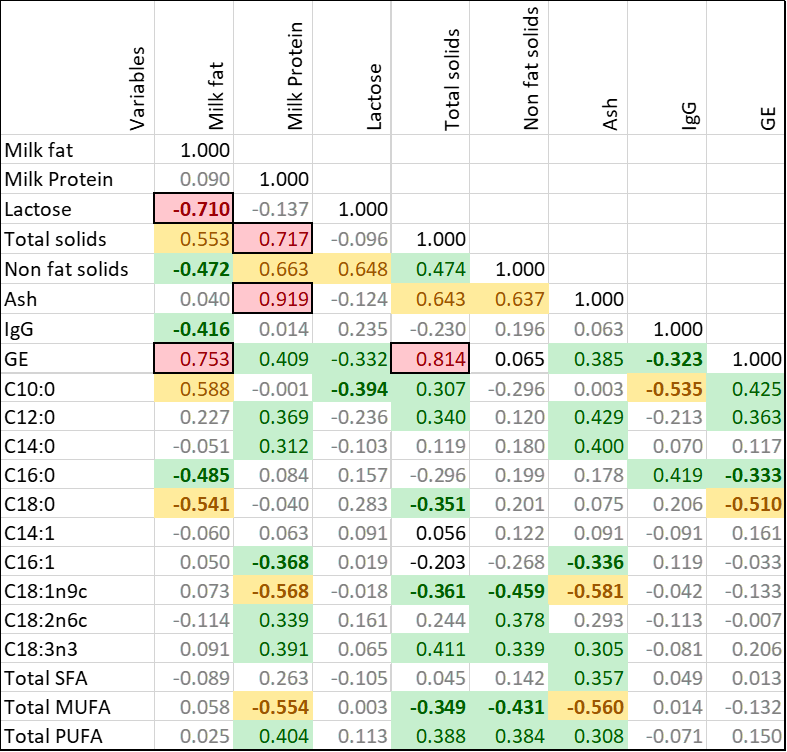

Supplement: S1 Table — Colored values are significant (p < 0.05) correlation coefficients (red: strong; yellow: medium; green: weak; in bold: negative). GE: gross energy; SFA: saturated fatty acids; MUFA: monounsaturated fatty acids; PUFA polyunsaturated fatty acids. (TIF) [file pone.0310693.s001.tif]

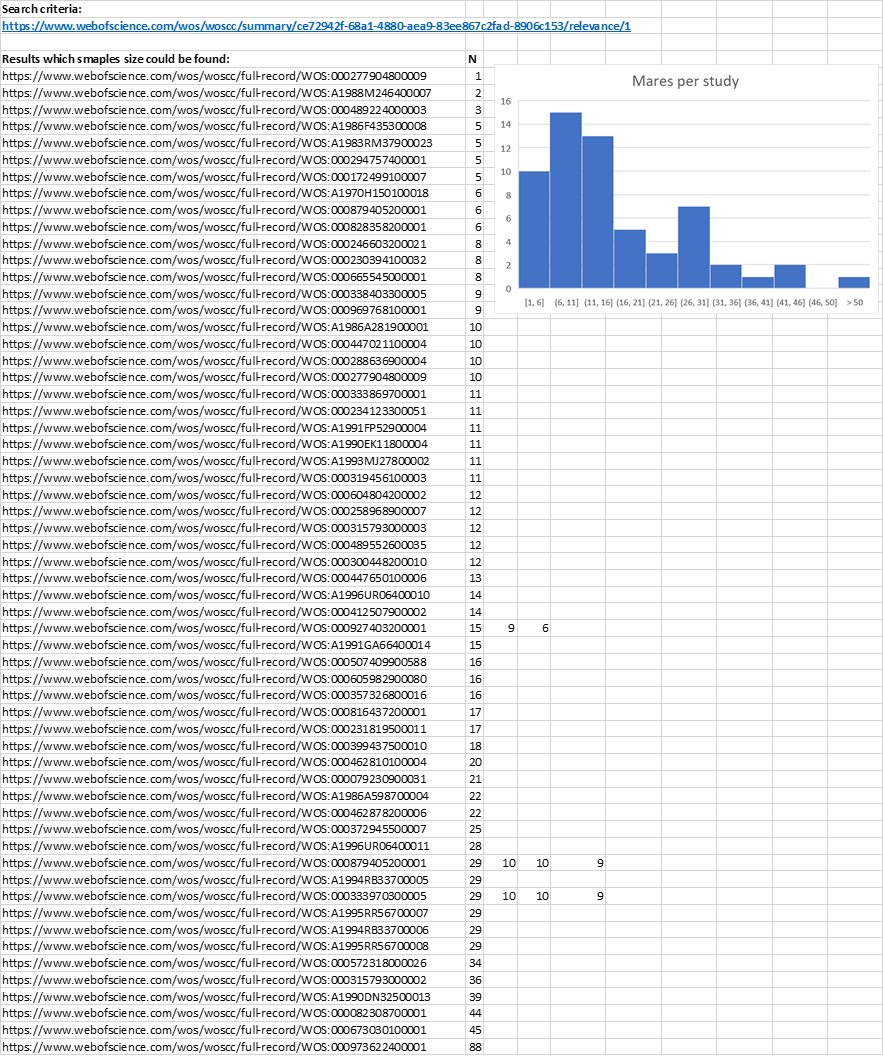

Supplement: S2 Table — N is number of mares sampled. (TIF) [file pone.0310693.s002.tif]
