# Supplementary material for: Nutritional composition, fatty acids profile and immunoglobulin G concentrations of mare milk of the Chilean Corralero horse breed
Source: PLoS One. 2024 Sep 19;19(9):e0310693. doi: 10.1371/journal.pone.0310693 (PMC11412646; doi:10.1371/journal.pone.0310693)
Supplement: Attachment — Submitted filename: Response to Reviewers.docx [file pone.0310693.s004.docx]

PONE-D-24-22129

Nutritional composition, fatty acids profile and immunoglobulin G concentrations of mare milk of the Chilean Corralero horse breed

PLOS ONE

**Journal requirements:**

1. When submitting your revision, we need you to address these additional requirements.

<https://journals.plos.org/plosone/s/file?id=wjVg/PLOSOne_formatting_sample_main_body.pdf> and 
<https://journals.plos.org/plosone/s/file?id=ba62/PLOSOne_formatting_sample_title_authors_affiliations.pdf>.

Answer: the format requirements were revised and addressed.

2. Thank you for stating the following financial disclosure: 
 [copy in funding statement].   Please state what role the funders took in the study.  If the funders had no role, please state: ""The funders had no role in study design, data collection and analysis, decision to publish, or preparation of the manuscript."" 
Answer: we have added the funders and their roles.

3. We note that Figure 1 in your submission contain copyrighted images.

Answer: This is not a copyrighted image, but an image provided by a co-authors’ family member.

4. We note that Figure 2 in your submission contain [map/satellite] images which may be copyrighted.

Answer: we have replaced the Google map for a new map. The map was created using QGIS 3.28.1 using a basemap called ESRI (Environmental Systems Research Institute) World Imagery Clarity. I have seen multiple PLOS ONE papers using ESRI maps and the detail of the website is below. We have now added the recommended caption below

The following uses are permitted:

- Personal use, internal business use, or to include in a presentation or a report for a client.
- In brochures and marketing collateral, or on a company website to promote your own products and services and display your store locations.
- In academic publications (for example, research journals, textbooks, and so on).
- In government works, so long as the government agency clearly delineates between government works that are in the public domain and third party works that are protected by copyright.
- **The following attribution is recommended as a caption to the image:** Map image is the intellectual property of Esri and is used herein under license. Copyright © 2020 Esri and its licensors. All rights reserved.

5. Review Comments to the Author

**Reviewer #1:** I'm sorry, but in my opinion the paper entitled Nutritional composition, fatty acids profile and immunoglobulin G concentrations of mare milk of the Chilean Corralero horse breed has serious problems in order to be accepted. The paper has basic methodological problems that make it difficult to publish in its current form.

Answer: We appreciate the time and effort placed in assessing our manuscript. We understand the concerns the reviewer has expressed, and along with the comments and suggestions received from the other two reviewers, we believe the manuscript has improved substantially. We have now added relevant information that was missing in the original submission and clarified several aspects that may have been misunderstood.

A work that aims to take into consideration the different factors that vary in milk quality (feeding, DIM, etc) should take into consideration sampling done in a more adequate way (sampling of the forage ingested and not the one available).

Answer: As stated at the end of the Introduction section, the objective of this study was to *analyse* the nutritional composition, fatty acid profile, and IgG concentration of the milk produced by Chilean Corralero horse mares (Fig. 1) from breeding farms located in southern Chile. We agree that if the objective were to analyse the effect of different environmental and management factors on mare milk composition, we should have designed an appropriate study to scientifically assess them. However, our aim was solely to characterise the milk produced by this horse breed and to explain some of the variation found across the three most important breeding farms in Chile. Thus, we have now changed 'analyse' to 'characterise'.

Furthermore, the statistical model adopted in my opinion is not adequate to evaluate the differences assessed.

Answer: We used a variety of descriptive statistics and statistical tests to accurately characterise the different factors and variables. For example, we used descriptive statistics to analyse the mares sampled (Figure 3). To determine if there were differences among farms in terms of categorical variables, we conducted a Chi-square analysis. It can be argued that the categories we used for birth number (1-3 vs 4+) and age (4-9 vs 10+) were arbitrary, and that we could have kept the variables as numeric and quantitative. We addressed the latter by conducting an unbalanced ANOVA, with farm and sex of the offspring as factors, and DIM and age as response variables. Furthermore, both tests yielded similar conclusions, indicating that neither age nor birth number varied with the farm. Given the numerous milk components (variables) considered, a multivariate approach is recommended to reduce the number of variables and found overall associations and trends amongst variables. General linear models were used to compare different factors (farm, age, birth no, sex f the offspring and DIM) on the response variables of interest. Finally, association between variables was assessed with correlations analysis. Nevertheless, we will be happy to consider any specific suggestion for statistical tests from the reviewer in a future iteration.

Furthermore, there is no reference in the materials and methods how milk fat was quantified. Furthermore, the forage analysis lacks the evaluation of the ethereal extract.
Again, why do the authors report the evaluation of milk quality according to the sex of the foal? What is the influence of this factor?

Answer: We apologies for overlooking this relevant aspect of the milk analysis. We have now added the protocol used to determine milk fat content to the Materials and Method section (under ‘Milk components determination’ heading). It was determined by the Gerber method (INN, 1998), using a calibrated butyrometer (Boeco,V1070, Hamburg, Germany), with 10 mL of H_2_SO_4_ (concentration 90–91% and density 1.818 ± 0.003 g mL-1 at 20ºC), 10.77 mL of the milk and 1 mL of isoamyl alcohol (2-methyl-1-butanol). The butyrometer was stirred and centrifuged at 1100 ± 100 rpm (diameter 500 ± 25 mm) for 4 - 5 min. Then, the butyrometer was subjected to 65°C for 5 min in a thermoregulated bath. The fat content was directly read via a calibrated butyrometer (Boeco,V1070, Hamburg, Germany).

Concerning EE analysis, the lipid content in forages is typically low and exhibits low variability, ranging between 2 and 3% of the DM (Glasser et al., 2023, DOI: 10.1016/j.anifeedsci.2013.06.010). Additionally, the forage components that have been shown to impact dry matter intake and energy consumption are crude protein (CP) and neutral detergent fibre (NDF) (Edouard et al., 2008, DOI: 10.1017/S1751731108002760). These components are routinely analysed in forages, in contrast to EE analysis. Therefore, while obtaining EE data would have been advantageous, it is not essential for characterising the forage offered to mares.

Regarding the foal's sex, studies (e.g., Barłowska et al., 2023) have shown that mares with female offspring produce milk with higher fat and protein concentrations and lower lactose content compared to mares with male offspring. We considered the foal's sex as a potential factor to explain some of the variations between farms: 'the only milk component affected by sex of the offspring was GE. The combined effect of a higher female offspring proportion and higher milk fat content of  mares of farm C would explain the higher GE values compared with farm B.'.

**Reviewer #2:** Manuscript PONE-D-24-22129, entitled “Nutritional composition, fatty acids profile and immunoglobulin G concentrations of mare milk of the Chilean Corralero horse breed”

This review article provides information about the nutritional composition, fatty acids profile and immunoglobulin G concentrations of mare milk of the Chilean Corralero horse breed. It is in general appropriately organized, carried out and written, however there are some points that should be corrected or clarified.

Answer: Thank you for reviewing our manuscript and providing valuable feedback to help us improve it. We have addressed your comments and suggestions, as well as those from the other two reviewers, resulting in substantial improvements to the manuscript. We hope the reviewer finds the changes made satisfactory.

L24: “The objective of the present study was to analyze…”

Answer: addition made.
L33-34: “…mares' milk were within the reported range for other horse breeds but tended to have lower levels for fat and total…”

Answer: change made.
L36: Please delete “effect”

Answer: deleted.
L43: “Milk of Mare (Equus caballus)…

Answer: change made.
L54: “…found to play a protective role against certain illnesses…”

Answer: addition made.
L56: “Mare and human milk…”

Answer: change made.
L60: “help lowering”

Answer: change made.
L64-65: “Additionally, mare milk is characterized by the ability to protect…”

Answer: change made.
L76: “…and Shetland (17) in terms of milk solids…”

Answer: a change made in line with the reviewers’ suggestion.
L88: What do you mean by “Rodeo symbolizes traditional cattle-working”?

Answer: this has now been changed to ‘Rodeo is based on the practical, real-life work that cowboys have to cull a steer from the herd.’
L101: Please provide the number of approval

Answer: We followed the guidelines of the Committee for the Ethical Use of Animals in Experiments but a specific approval was not required since we did not perform any regulated procedures (e.g., blood sampling); the animals were submitted only to routine farm practices. This is in line with the Chilean legislation. Thus, we have now included the following statement: ‘*All the procedures, including animal care and handling procedures, followed national legislation (Law No. 20,380 on Protection of Animals; Decree No. 29 about regulation on the protection of animals during their industrial production, their commercialization and in other areas to hold animals), whose application is supervised by the National Service of Agriculture and Livestock (SAG), the competent authority in this matter*.’
L105: Fig. 2?

Answer: Thanks for spotting this issue. We had swapped figures numbers in our original submission. Now Fig 2 is the map and Fig 3 is the infographic.

L109: Please check figure and correct

Answer: Figure 3 is the correct figure to cite in this line (now L111).

L111: Fig. 2 or 3?

Answer: Figure 2.
L119: Fig. 3?

Answer: Now Figure 2 (map with location of the farms).
L139-140: What do you mean by “a more regular air temperate”?

Answer: this has now been removed.
L149: “Samples were aseptically collected from the mares, at a volume of 250 mL per…”

Answer: correction made.
L154: “collected” instead of “taken”

Answer: change made.
L163: “sampled” instead of “taken”

Answer: change made.
L164: “removed” instead of “taken”

Answer: a change was made in line with the reviewers’ suggestion.
L247: “among” instead of “between”

Answer: change made.
L249: These results are not shown in Table 2

Answer: No, they are not shown otherwise we would be repeating the same information in the table and text. Table 2 shows that there are differences among farms while in these lines we provide the actual means for each farm (not shown in table 2).
Table 2: What about the Farm (B-C)?

Answer: Given that farm B and C were very similar in DIM and the mean milk components, we simplified the table by not presenting this GLM result since it would be redundant.
L266: What do you mean by “(Error! Reference source not found.4)”?

Answer: The original Word document shows ‘Fig. 4’ instead. There must be an error of the system when converted the docx file into a PDF.
L270: “…but greater lactose content (Fig. 4).”

Answer: information added.
L283: “affecting” instead of “driving”

Answer: change made.
L283: Please add the number of Table in the parenthesis

Answer: the table number (2) is shown in the original Word document. The automatic conversion into a PDF must have removed it.
L285-286: Where are these data shown?

Answer: they are shown in the supporting information file. Now added.
L290: The former or both?

Answer: ‘both’ is correct. Correction made.
L300: Inverse or negative?

Answer: they have the same meaning; a negative correlation is an inverse association, while a positive correlation is a direct association.
L216: “Among these studies…”

Answer: change made.
L323-326: This sentence is not necessary. How were these percentages calculated?

Answer: with this sentence, we aim to demonstrate that our study is among the top 5-10% in terms of the number of samples used to characterize mare milk. The relevant information used to calculate these percentages can be found in the supplementary material, specifically in the provided Table S2.
L331: “Furthermore” instead of “More so”

Answer: change made.
L339: The duration of the gestation is a characteristic that is varied within breed and can be manipulated by the farmer?

Answer: what we meant to imply is that the different farms can have different breeding management, hence the foaling date can vary, and thus the DIM among farms can vary. The other reason for a difference in DIM could be the gestation length.
L358-361: It is confounding as it is written

Answer: the sentence was split and re-phrased to improve clarity.
L370-371: “…suggesting that there are other factors than DIM imposing variation between the three examined breeding farms. This…”

Answer: change made.
L395: “…composition in horses.”

Answer: addition made.
L412: “…how they vary with cow…”

Answer: change made.
L415: Please add a reference

Answer: change made.
L419: Please delete “of”

Answer: deleted.
L425: “Lowe et al. (9) apart from Haflinger and Shetland Pony, and greater fat levels than that of Dutch Saddle…”

Answer: change made.
L432: “except for farm B”

Answer: change made.
L443: “observed” instead of “seen”

Answer: change made.
L448: In L444, you state that SFA prevail.

Answer: UFA = PUFA + MUFA. In our study, UFA surpassed SFA.
L452: “…horse milk has a low stearic…”

Answer: ‘exhibits’ is equally correct, only maybe a little bit more sophisticated than ‘has’.
L454: “high” instead of “heigh”

Answer: correction made.
L464: “…and 26.8 mg/g DM, respectively (77).”

Answer: addition made.
L466: “…analyzed in our study contained these fatty acids.”

Answer: addition made.
L479: “farm” instead of “fam”

Answer: correction made.
L496: “…age has also been previously observed in horses (83).”

Answer: change made.
L517: “would make sense”?

Answer: changed to ‘would be expected.’
L531: “sustain” instead of “see”

Answer: correction made in line with reviewer’s suggestion.
L549: “one of the top-ranking studies in this field”?

**Reviewer #3:** General opinion
In this study evaluated the various factors on nutritional composition and immunological properties of milk from the Chilean Corralero horse breed and discussed its potential implications for foal health and human consumption. The number of samples are very remarkable, this ensures the reliability of the results. The aim of this manuscript is very interesting for the readers; however, this manuscript must be improve, mainly the Results section.

Answer: Thank you very much for your positive remark. We have addressed your comments and those from the other two reviewers. We believe the manuscript has been enhance with the reviewers’ valuable input.

Detailed opinion:
line 117: please add the amount of precipitation!

Answer: information added for the three locations.
lines 130-131: the information in the brackets are better placed if put into the farm „A”!

Answer: change made.
Table 2 and 3: recommendation: all mean values of parameters would be shown in Table 2 and 3, not only overall mean and results of GLM!

Answer: we did consider this option, of course. However, we decided against including this information in order to not focus on the farm averages, but rather to give an overall characterization of the milk from the horse breed. The statistical analysis was conducted to help explain the observed variation.
line 250: MUFA is only found in Table 3!

Answer: the information that supports this sentence is in the Supporting information Table S1. This has now been added.
line 266: mistake!

Answer: The original Word document shows ‘Fig. 4’ instead. There must be an error of the system when converted the docx file into a PDF.
line 283: missing the number of a Table!

Answer: the table number (2) is shown in the original Word document. The automatic conversion into a PDF must have removed it.
Figure 5: recommend inserting to the Figure a headline for better meaning!

Answer: we believe the figure caption is self-explanatory ‘Fig. 5 – Pearson’s correlation of fatty acid concentrations. Circle size represents strength of correlation and color represents correlation r-value (-1.0 to 1.0). Values in white squares are r-values with p-values below.’
